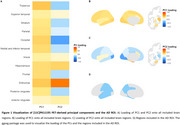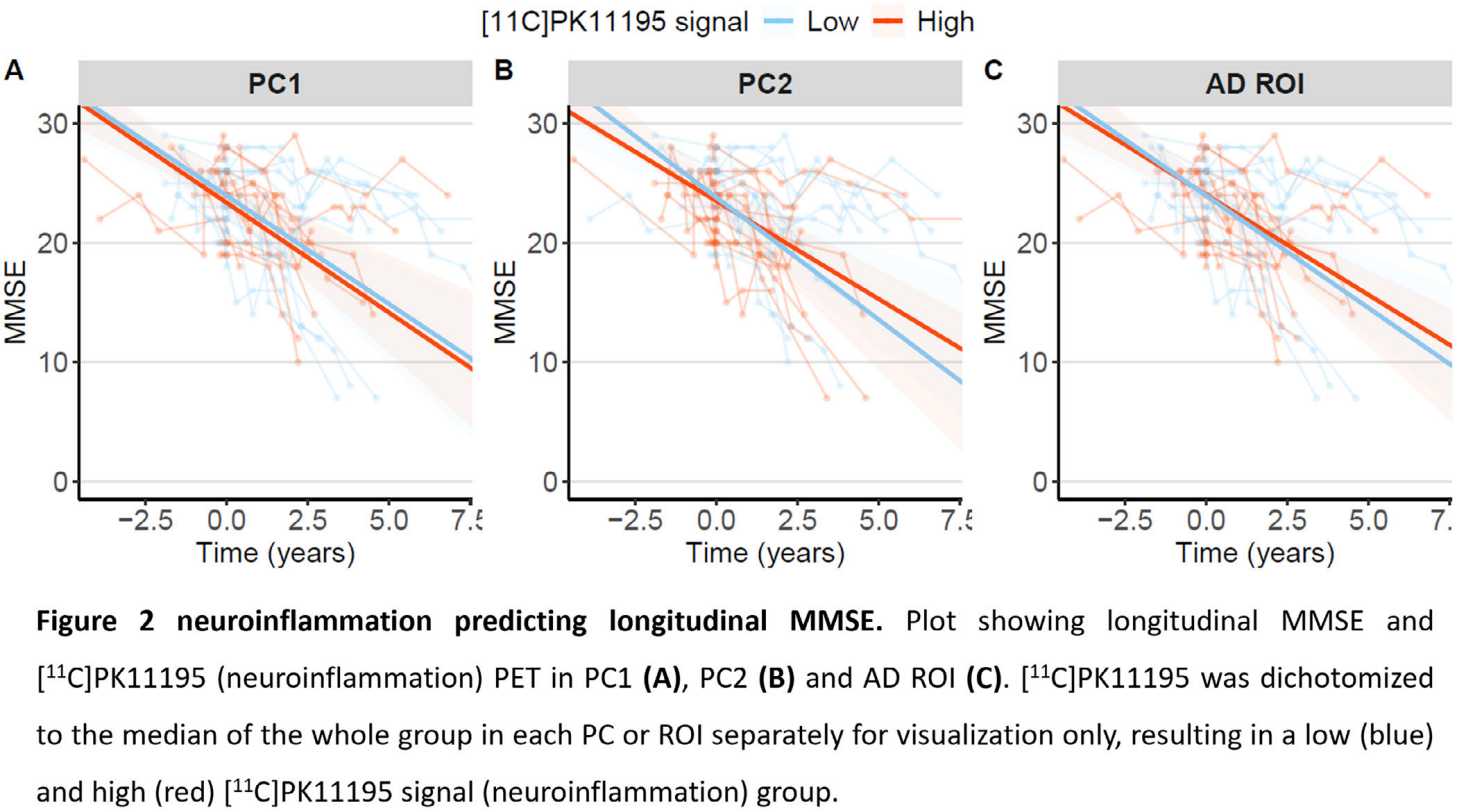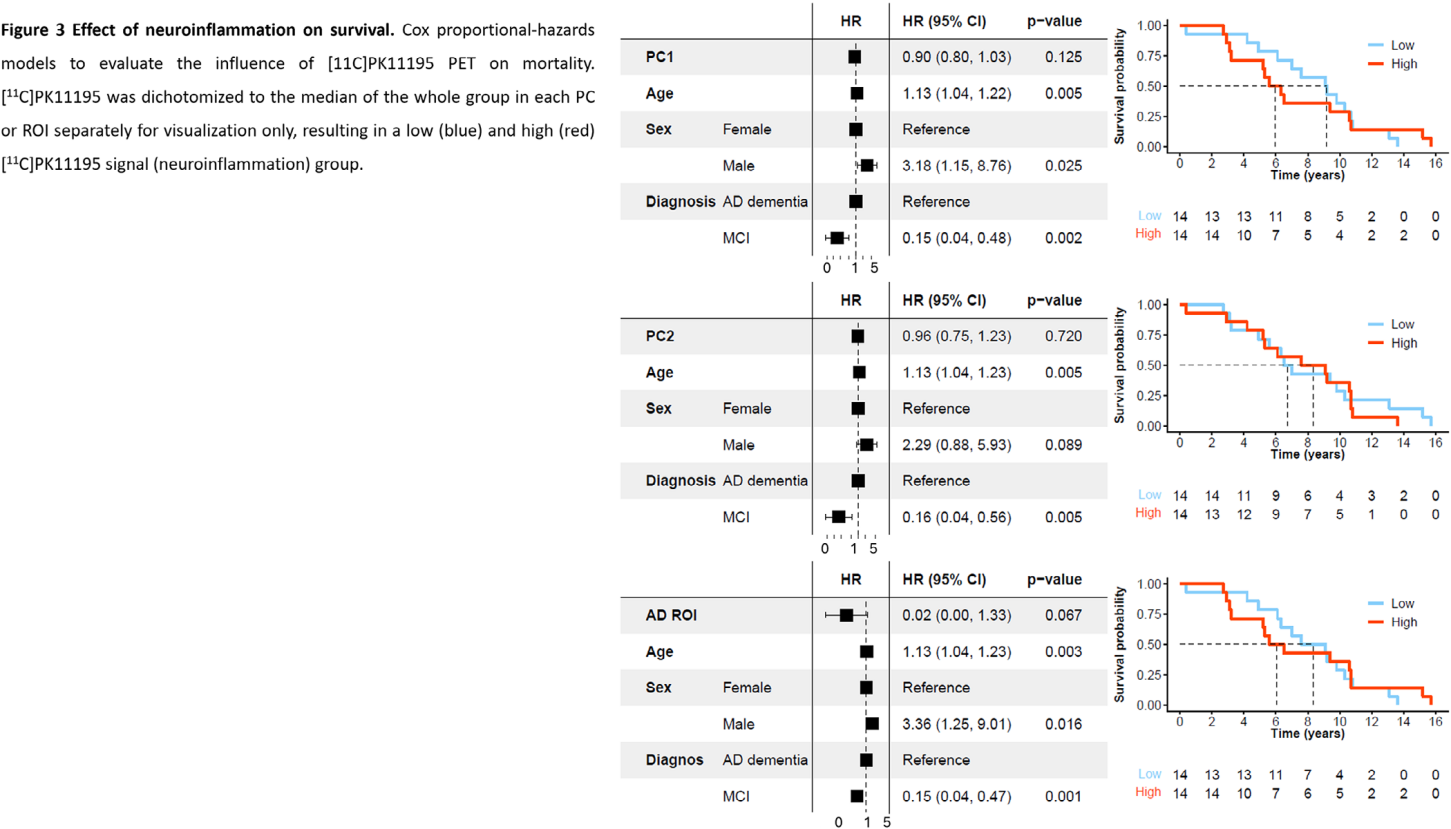# Prognostic value of neuroinflammation [^11^C]PK11195 PET on longitudinal cognitive decline and survival up to 15 years after PET in Alzheimer’s disease

**DOI:** 10.1002/alz70861_108534

**Published:** 2025-12-23

**Authors:** Roos M. Rikken, Maqsood Yaqub, Emma M. Coomans, Ellen Dicks, Anne E. van der Vlies, Frederik Barkhof, Albert D. Windhorst, Yolande A.L. Pijnenburg, Wiesje M. van der Flier, Ronald Boellaard, Everard G.B. Vijverberg, Elsmarieke van de Giessen

**Affiliations:** ^1^ Amsterdam Neuroscience, Brain Imaging, Amsterdam Netherlands; ^2^ Alzheimer Center Amsterdam, Neurology, Vrije Universiteit Amsterdam, Amsterdam UMC location VUmc, Amsterdam Netherlands; ^3^ Radiology & Nuclear Medicine, Vrije Universiteit Amsterdam, Amsterdam UMC location VUmc, Amsterdam Netherlands; ^4^ Amsterdam Neuroscience, Neurodegeneration, Amsterdam Netherlands; ^5^ Department of Neurology, Alzheimer Center Amsterdam, Amsterdam Neuroscience, Vrije Universiteit Amsterdam, Amsterdam Netherlands; ^6^ Amsterdam Neuroscience, Neurodegeneration, Amsterdam, Noord‐Holland Netherlands; ^7^ Department of Radiology and Nuclear Medicine, Vrije Universiteit Amsterdam, Amsterdam UMC location VUmc, Amsterdam Netherlands; ^8^ Queen Square Institute of Neurology and Centre for Medical Image Computing, University College London, London, Greater London UK; ^9^ Alzheimer Center, Amsterdam University Medical Center, location VUmc, Amsterdam, Noord‐Holland Netherlands; ^10^ Department of Epidemiology and Data Science, Vrije Universiteit Amsterdam, Amsterdam UMC, Amsterdam, North Holland Netherlands; ^11^ Department of Radiology and Nuclear Medicine, Vrije Universiteit Amsterdam, Amsterdam University Medical Center, location VUmc, Amsterdam Netherlands

## Abstract

**Background:**

Neuroinflammation plays a key role in Alzheimer’s disease (AD) pathophysiology. However, whether neuroinflammation has a prognostic effect on disease progression is largely unknown. Therefore, we aim to investigate the role of neuroinflammation as measured using TSPO PET on longitudinal cognition and survival.

**Method:**

We included 28 amyloid‐positive participants (*N* =9 MCI, N=19 AD dementia) and 21 healthy controls from a historical cohort who underwent dynamic [^11^C]PK11195 (TSPO) PET to quantify neuroinflammation. Principal component analysis was performed to identify relevant [^11^C]PK11195 signal (Figure 1). An additional AD ROI consisting of temporal and parietal regions was investigated. Longitudinal MMSE covering a period up to 11 years was used to measure cognitive decline (mean time: 4.35, range: 0.1‐11.7 years). We used linear mixed models corrected for age, sex and diagnosis to investigate the effect of neuroinflammation PET on cognition both in MCI and AD combined and within both diagnostic groups separately. Survival data were available for all participants, up to 15.7 years (median: 7.3, range: 0.4‐15.7 years) after PET. To examine the influence of neuroinflammation on survival time, we used age, sex, and diagnosis adjusted cox proportional‐hazards models.

**Result:**

Two principal components (PC) explaining >10% of variance were retained. PC1 explained 55.4% of the variance and was most explained by [11C]PK11195 binding in the thalamus and entorhinal cortex. PC2 explained 15.3% of the variance and loaded mostly onto the entorhinal cortex (Figure 1). [^11^C]PK11195 in PC1 or PC2 did not predict longitudinal MMSE (PC1: β=‐0.03, *p* =0.963; PC2: β=0.123, *p* =0.335; AD ROI: β=1.3, *p* =0.571) or survival (PC1: HR=0.90, *p* =0.13; PC2: HR=0.96, *p* =0.7) (Figure 2). Higher [11C]PK11195 PET in the AD ROI showed a non‐significant trend at being associated with reduced survival PC1 (HR=0.02 [0.00, 1.33], *p* =0.06) (Figure 3). Interestingly, we found a significant effect of the PCs and AD ROI on longitudinal MMSE in MCI, (PC1: β=0.06, *p* =0.014; PC2: β=0.123, *p* <0.001; AD ROI: β=2.1, *p* =0.008) but not in AD (PC1: β=0.06, *p* =0.474; PC2: β=0.22, *p* =0.117; AD ROI: β=1.7, *p* =0.544).

**Conclusion:**

We found preliminary evidence for neuroinflammation PET to predict longitudinal cognition in MCI specifically, but not in AD or AD and MCI combined.